# Course of depressive symptoms in men and women: differential effects of social, psychological, behavioral and somatic predictors

**DOI:** 10.1038/s41598-019-55342-0

**Published:** 2019-12-12

**Authors:** Ana N. Tibubos, Elmar Brähler, Mareike Ernst, Carlotta Baumgarten, Joerg Wiltink, Juliane Burghardt, Matthias Michal, Jasmin Ghaemi Kerahrodi, Andreas Schulz, Philipp S. Wild, Thomas Münzel, Irene Schmidtmann, Karl J. Lackner, Norbert Pfeiffer, Andreas Borta, Manfred E. Beutel

**Affiliations:** 1 Department of Psychosomatic Medicine and Psychotherapy, University Medical Center of the Johannes Gutenberg-University Mainz, Mainz, Germany; 2 DZHK (German Center for Cardiovascular Research), partner site Rhine-Main, Mainz, Germany; 3 Preventive Cardiology and Preventive Medicine - Center for Cardiology, University Medical Center of the Johannes Gutenberg-University Mainz, Mainz, Germany; 4 Center for Cardiology - Cardiology I, University Medical Center of the Johannes Gutenberg-University Mainz, Mainz, Germany; 5 Institute of Medical Biostatistics, Epidemiology and Informatics, University Medical Center of the Johannes Gutenberg-University Mainz, Mainz, Germany; 6 Institute of Clinical Chemistry and Laboratory Medicine, University Medical Center of the Johannes Gutenberg-University Mainz, Mainz, Germany; 7 Department of Ophthalmology, University Medical Center Mainz of the Johannes Gutenberg-University Mainz, Mainz, Germany; 8 Center for Thrombosis and Hemostasis, University Medical Center of the Johannes Gutenberg-University Mainz, Mainz, Germany; 9 Boehringer Ingelheim Pharma GmbH Co KG, Ingelheim am Rhein, Germany

**Keywords:** Public health, Epidemiology

## Abstract

In this study, we aimed to identify the most important and sex-specific social, psychological, behavioral and somatic predictors of recurrent depressive symptoms. Data was obtained at two measurement points within five years by the Gutenberg Health Study (GHS). Out of N = 12,061 individuals, a sample of 877 (age 52.3 ± 9.9) who reported clinically relevant depressive symptoms at baseline was analyzed. Univariate analyses and multiple logistic regression analyses were conducted. Almost half of participants depressed at baseline also reported depressive symptoms five years later. Sex-stratified multivariate analyses revealed that solely social support remained a significant protective predictor against recurrence of depression in men (OR = 0.93; CI_95%_ = 0.87–0.99), whereas in women smoking (OR = 1.97; CI_95%_ = 1.23–3.22), and Type D personality (OR = 1.65; CI_95%_ = 1.10–2.49) were significant risk factors. However, when analyzing the entire sample, no interaction effect between sex and each predictor turned out to be significant. Only social support was retained as an overall predictive factor. As depressive symptoms recur, depressive vulnerability is established involving personality, health behavior and social factors. Although no significant sex-specific interactions were observed, sex-stratified analyses point out different patterns for relevant predictors of recurrent depressive symptoms in men and women.

## Introduction

Depression is one of the most debilitating mental disorders in the general population. Persistent depressive symptoms in particular are among of the strongest predictors of reduced quality of life, work disability, unhealthy life style, cardiovascular disease^1^ increased health care use, and even premature death^2^. In an elderly Canadian sample^3^ as many as 55% of participants experienced recurrence at some point over a 6 year follow-up after an major depressive disorder (MDD) episode. According to the systematic review of Hardeveld, *et al*.^4^, recurrence of MDD was considerably higher in patients of specialized mental health care settings (60% after 5 years, 67% after 10 years and 85% after 15 years) than in the general population (35% after 15 years). In the Netherlands Study of Depression and Anxiety (NESDA), 47.6% of patients with depressive disorder at baseline but only 25.1% of the combined depression and anxiety group were without a disorder at two year follow-up^5^. Systematic reviews found that childhood maltreatment, larger numbers of previous depressive episodes and residual symptoms increased the risk of recurrence along with longer duration of the depressive episode^6,7^. In the latest systematic review^7^ only five cohorts from two countries were identified investigating risk factors of recurrent depression: These are NESDA and The Netherlands Mental Health Survey and Incidence Study (NEMESIS) in Europe, in the USA the Collaborative Depression Study (CDS), the Group Health Cooperative (GHC) and the National Epidemiological Survey on Alcohol and Related Conditions (NESARC) with a full range of psychiatric characteristics, biological, genetic and neuroimaging variables as potential risk factors for recurrent depression. However, psychosocial measures such as loneliness and perceived social support were only focused in the Dutch studies which are in particular interest for psychotherapy interventions. Given the scarcity of longitudinal and of community-based studies, there is a strong clinical need for reliable prognostic factors treatment of patients prone to developing a persistent course. Besides comorbidity with other mental disorders, mostly anxiety disorders^5^, sociodemographic, psychological, somatic, and behavioral factors have been conceptualized as risks in the vulnerability-stress model^8^ to develop a persistent course of depression.

Epidemiological studies point to a female preponderance in the development of depression during their life-time, however, findings regarding gender differences in the course of depression have been controversial^9^. While sex usually refers to a biological construct, rooted in genetics, anatomy, and physiology, psychosocial and behavioral variables characterizing men and women are subsumed under gender^10^. Although studies include sex as predictor or confounder by default, gender-sensitive analyses according to, for instance, guidelines provided by the National Institutes of Health (NIH) in the USA, Canada^11^ and the Robert Koch Institute (RKI) in Germany^12^ have been neglected in most cases.

*Sociodemographic factors* beyond sex such as younger age at onset^6,13^ as well as current older age^5^ have been identified as predictors for chronic depression. In a meta-analysis, low socioeconomic status increased the odds of persistent depression^14^. Colman, *et al*.^3^ however found no effect of demographic variables on recurrent depression in multivariate analyses taking into account smoking, history of depression and mastery.

*Psychological and psychosocial determinants* were significant predictors of the course of depression including a family history of mood disorders^6^. First onsets of depression more frequently occurred following major stressful life events in patient samples than without such an event. However, this was not the case in community samples for recurrent depressive episodes^14^. Distressed personality, with its facets negative affectivity and social inhibition, was associated with increased vulnerability to depressive disorders and symptoms^15^. While neuroticism increased the likelihood of recurrent depression, extraversion, agreeableness, conscientiousness and a large network size reduced the risk of a new episode of depression^16^. Social support had protective effects over the life span^17^. In a twin study, women reported higher levels of social support than men and only in women did it reduce the risk of subsequent depression^18^.

Depression has also been related to *behavioral factors*, such as physical inactivity, and increased consumption of tobacco and alcohol. In individuals with major depression, current smoking^3^, alcohol abuse, and physical inactivity carried an increased risk of recurrent depression^19^. Chronic somatic diseases as *somatic factors* were linked to higher likelihood of depression^1^. However, it has remained inconclusive whether somatic diseases are predictive for recurrent depression^8,19^. Evidence regarding sex-specific behavioral and somatic determinants of the course of recurrent depression has been lacking to date.

Based on a unique, comprehensive data set of a large longitudinal population-based study, we analyzed the effects of sociodemographic, psychological, behavioral, and somatic baseline data on incident depressive symptoms five years later. By sex-sensitive evaluation, provided through sex-stratified results presentation and modelling interactions with sex as moderator as proposed by health institutes, e.g. NIH or RKI, we tried to disentangle gender-related mechanisms of trajectory of depressive symptoms. We took care to include participants who were depressed at baseline, and who may or may not have reported a previous diagnosis of depression. Our aims were:

1. to investigate the prevalence of depressive symptoms in men and women five years after baseline who had previously shown evidence of depression
2. to determine social, psychological, behavioral and somatic predictors of recurrent depressive symptoms among men and women
3. to test sex-specific differences in these predictors of recurrent depressive symptoms.

## Results

### Differential effects of social, psychological, behavioral and somatic predictors of recurrent depressive symptoms

Table 1 depicts univariate analyses of depressed participants at baseline according to the presence of depressive symptoms. Presented are baseline sociodemographic, psychological, health-behavior measures, somatic diseases, and antidepressant intake. Among sociodemographic factors, lower SES significantly predicted higher rates of subsequent depressive symptoms, but no significant associations with age or partnership status were observed. Sex failed to reach significance despite of higher amount of women with recurrent depressive symptoms compared to men. With regard to health-related behaviors, only smoking showed significant associations with depressive symptoms. No relation was found between somatic disorders and recurrent depression. Also, no effect was found for life events in the previous year. In contrast, participants with clinically relevant depressive symptoms reported less perceived social support. Anxiety disorders, Type D personality, loneliness, lower social support, generalized anxiety, social phobia, panic and intake of antidepressants were associated with elevated depressive symptoms at follow-up. As to be expected by definition, those who also reported a history of depression were more likely to report recurrence of depressive symptoms. Hence, in multivariate analysis, medical history of a lifetime diagnosis of depression was excluded as predictor from the model to avoid tautological reasoning.

**Table 1:** Descriptive baseline data for recurrent depressed participants according to presence of clinically elevated depressive symptoms (PHQ9 ≥ 10) at five year follow-up (FU) later (N = 877).

	All (*N* = 877)	No depression (*N* = 453, 51.65%)	Depression at FU (*N* = 424, 48.35%)	p
***Sociodemographic***				
Sex (women, %)	59.6 (523)	56.7 (257)	62.7 (266)	0.074
Age (years)	52.3 ± 9.9	52.6 ± 10.1	52.0 ± 9.7	0.310
**SES**	12.36 ± 4.18	12.67 ± 4.37	12.04 ± 3.96	**0.026**
Living with partner (%)	68.9 (604)	70.2 (318)	67.5 (286)	0.380
***Behavioral***				
**Smoking** (%)	25.4 (223)	22.3 (101)	28.8 (122)	**0.030**
Active sports (%)	42.9 (376)	43.9 (199)	41.7 (177)	0.540
Alcohol above tolerance (%)	21.0 (184)	19.2 (87)	22.9 (97)	0.190
***Somatic***				
CVD (%)	12.5 (110)	12.6 (57)	12.5 (53)	1.000
Cancer (%)	8.1 (71)	7.8 (35)	8.5 (36)	0.710
Diabetes (%)	9.5 (83)	10.4 (47)	8.5 (36)	0.360
Obesity (%)	30.7 (269)	28.5 (129)	33.2 (140)	0.140
***Psychological***				
**Type D personality** (%)	58.3 (509)	51.4 (232)	65.6 (277)	**<0.001**
Life events (last 12 months)	3.66 ± 3.13	3.73 ± 3.14	3.59 ± 3.12	0.510
**Loneliness** (%)	36.7 (318)	30.6 (137)	43.1 (181)	**<0.001**
**Social support**	17.73 ± 4.26	18.13 ± 4.29	17.31 ± 4.19	**0.005**
**Generalized Anxiety** (%)	46.7 (408)	39.8 (179)	54.1 (229)	**<0.001**
**Social phobia** (%)	33.3 (290)	27.2 (122)	39.8 (168)	**<0.001**
**Panic** (%)	24.3 (203)	21.0 (90)	27.8 (113)	**0.024**
**History of depression** (%)	47.3 (413)	38.6 (175)	56.5 (238)	**<0.001**
**History of anxiety disorder** (%)	25.3 (221)	22.3 (101)	28.5 (120)	**0.036**
***Medication intake***				
**Antidepressant** (%)	22.0 (192)	17.2 (77)	27.1 (115)	**<0.001**

*Note*: PHQ-9 = Patient Health Questionnaire-9; SES = socioeconomic status; CVD = cardiovascular risk disease; Significance tests: For mean ± standard deviation like 52.3 ± 9.9 the t-test was used. The Chi-square test was used for frequencies like 59.6 (523).

Descriptive and inference statistics stratified for men and women are displayed in Table 2. Lower SES among recurrent depressed was only significant in male, not in female participants. With regard to health-related behavior smoking was only significantly linked with recurrent depressive symptoms among females, along with a trend for increased alcohol use. Somatic diseases were not linked with depressive symptoms at follow-up. Type D personality, comorbid anxiety disorders, and antidepressant intake were linked with recurrent depressive symptoms for both sexes in univariate analyses. Additionally, loneliness and social support were significant predictors, but only for men.

**Table 2:** Descriptive overview separately for men and women with recurrent depressive symptoms (PHQ9 ≥ 10) at follow-up (FU) five years later with inference statistics.

	Men				Women			
	All (*N* = 354)	No depression (*N* = 196, 55.37%)	Depression at FU (*N* = 158, 44.63%)	p	All (*N* = 523)	No depression (*N* = 257, 49.14%)	Depression at FU (*N* = 266, 50.86%)	p
***Sociodemographic***								
Age (years)	52.4 ± 9.4	52.6 ± 9.8	52.3 ± 9.0	0.790	52.2 ± 10.2	52.7 ± 10.3	51.8 ± 10.1	0.300
**SES**	13.26 ± 4.31	13.69 ± 4.40	12.71 ± 4.15	**0.033**	11.76 ± 3.98	11.88 ± 4.18	11.64 ± 3.80	0.500
Living with partner (%)	73.7 (261)	76.0 (149)	70.9 (112)	0.280	65.6 (343)	65.8 (169)	65.4 (174)	1.000
***Behavioral***								
**Smoking** (%)	29.1 (103)	28.1 (55)	30.4 (48)	0.640	22.9 (120)	17.9 (46)	27.8 (74)	**0.009**
Active sports (%)	36.2 (128)	36.7 (72)	35.4 (56)	0.820	47.4 (248)	49.4 (127)	45.5 (121)	0.380
Alcohol above tolerance (%)	23.2 (82)	23.0 (45)	23.4 (37)	1.000	19.5 (102)	16.3 (42)	22.6 (60)	0.080
***Somatic***								
CVD (%)	17.2 (61)	17.3 (34)	17.1 (27)	1.000	9.4 (49)	8.9 (23)	9.8 (26)	0.770
Cancer (%)	5.9 (21)	7.1 (14)	4.4 (7)	0.370	9.6 (50)	8.2 (21)	10.9 (29)	0.370
Diabetes (%)	11.9 (42)	11.7 (23)	12.0 (19)	1.000	7.9 (41)	9.3 (24)	6.4 (17)	0.260
Obesity (%)	33.3 (118)	32.1 (63)	34.8 (55)	0.650	29.0 (151)	25.7 (66)	32.2 (85)	0.120
***Psychological***								
**Type D personality** (%)	62.9 (222)	56.4 (110)	70.9 (112)	**0.006**	55.2 (287)	47.7 (122)	62.5 (165)	**<0.001**
Life events (last 12 months)	3.67 ± 3.27	3.68 ± 3.31	3.66 ± 3.22	0.940	3.66 ± 3.04	3.77 ± 3.00	3.55 ± 3.07	0.410
**Loneliness** (%)	34.1 (119)	25.9 (50)	44.2 (69)	**<0.001**	38.4 (199)	34.3 (87)	42.4 (112)	0.058
**Social support**	17.78 ± 4.17	18.45 ± 4.17	16.94 ± 4.03	**<0.001**	17.71 ± 4.33	17.89 ± 4.38	17.53 ± 4.28	0.350
**General Anxiety** (%)	42.7 (151)	35.7 (70)	51.3 (81)	**0.004**	49.5 (257)	42.9 (109)	55.8 (148)	**0.004**
**Social phobia** (%)	30.2 (106)	23.2 (45)	38.9 (61)	**0.002**	35.5 (184)	30.3 (77)	40.4 (107)	**0.017**
Panic (%)	21.1 (71)	18.9 (35)	23.7 (36)	0.350	26.5 (132)	22.5 (55)	30.2 (77)	0.055
**History depression** (%)	39.1 (138)	30.6 (60)	49.7 (78)	**<0.001**	52.8 (275)	44.7 (115)	60.6 (160)	**<0.001**
History anxiety disorder (%)	19.8 (70)	18.4 (36)	21.7 (34)	0.500	29.0 (151)	25.3 (65)	32.6 (86)	0.082
***Medication intake***								
**Antidepressant** (%)	17.0 (60)	13.3 (26)	21.5 (34)	**0.047**	25.4 (132)	20.2 (51)	30.5 (81)	**0.009**

*Note*: PHQ-9 = Patient Health Questionnaire-9; SES = socioeconomic status; CVD = cardiovascular disease. Significance tests: For mean ± standard deviation like 52.3 ± 9.9 the t-test was used. The Chi-square test was used for frequencies like 59.6 (523).

Logistic regression models of depressive symptoms at follow-up are displayed in Table 3 stratified by sex. Logistic regression models for men and women each revealed different patterns of predictors for recurrent depressive symptoms. For men, only social support remained a protective factor (OR = 0.93; 95% CI = 0.87 to 0.99) against a new episode of depressive symptoms. For the female population, smoking (OR = 1.97; 95%CI = 1.123 to 3.22), along with Type D personality (OR = 1.64; 95% CI = 1.20 to 2.27) were predictive risk factors.

**Table 3:** Multiple logistic regression model on recurrent depression (PHQ9 ≥ 10) for men and women separately.

	Men (*N* = 328; *N* = 148 events)						Women (*N* = 468; *N* = 241 events)					
	Odds Ratio	L (95%CI)	U (95%CI)	p	Standard error	z-value	Odds Ratio	L (95%CI)	U (95%CI)	p	Standard error	z-value
***Sociodemographic***												
Age [5 years]	0.948	0.818	1.097	0.480	0.075	−0.713	0.926	0.826	1.036	0.180	0.058	−1.340
SES	0.967	0.913	1.024	0.250	0.029	−1.140	0.980	0.929	1.034	0.460	0.027	−0.743
Living with partner	1.469	0.814	2.689	0.210	0.304	1.270	1.290	0.834	2.003	0.250	0.223	1.140
***Behavioral***												
**Smoking**	1.090	0.637	1.861	0.750	0.273	0.316	**1.974**	**1.227**	**3.215**	**0.006**	**0.245**	**2.770**
Active sports	1.048	0.631	1.741	0.860	0.258	0.180	0.944	0.636	1.403	0.780	0.202	−0.285
Alcohol above tolerance	1.082	0.623	1.877	0.780	0.281	0.281	1.575	0.935	2.675	0.090	0.268	1.700
***Somatic***												
CVD	1.093	0.553	2.165	0.800	0.347	0.257	1.127	0.552	2.309	0.740	0.363	0.330
Cancer	0.635	0.210	1.771	0.400	0.537	−0.847	1.501	0.753	3.047	0.25	0.354	1.150
Diabetes	0.995	0.432	2.052	0.890	0.396	−0.142	0.565	0.267	1.168	0.130	0.374	−1.530
Obesity	1.213	0.711	2.069	0.480	0.271	0.712	1.507	0.962	2.375	0.075	0.230	1.780
***Psychological***												
**Type D personality**	1.495	0.863	2.604	0.150	0.281	1.430	**1.651**	**1.100**	**2.485**	**0.016**	**0.208**	**2.410**
Life events (past 12 months) [per 5 events]	0.972	0.670	1.411	0.880	0.189	−0.149	0.823	0.583	1.154	0.260	0.174	−1.120
Loneliness	1.614	0.935	2.792	0.085	0.278	1.720	1.073	0.695	1.655	0.750	0.221	0.318
**Social support**	**0.931**	**0.871**	**0.994**	**0.034**	**0.034**	**−2.120**	0.981	0.934	1.031	0.460	0.025	−0.747
Generalized Anxiety	1.321	0.804	2.167	0.270	0.253	1.100	1.265	0.848	1.887	0.250	0.204	1.150
Social phobia	1.355	0.766	2.400	0.300	0.291	1.040	1.056	0.681	1.635	0.810	0.223	0.244
Panic	1.235	0.672	2.272	0.500	0.310	0.682	1.392	0.865	2.251	0.170	0.243	1.360
History of anxiety disorder	0.960	0.498	1.834	0.900	0.331	−0.123	1.061	0.667	1.686	0.800	0.236	0.250
***Medication intake***												
Antidepressant	1.477	0.740	2.968	0.270	0.353	1.100	1.581	0.992	2.536	0.055	0.239	1.920

*Note*: PHQ-9 = Patient Health Questionnaire-9; SES = socioeconomic status; CVD = cardiovascular risk disease; L CI = lower confidence interval; U CI = upper confidence interval.

### Sex-specific effects in the course of depressive symptoms

In order to test sex-specific differences in social, psychological, behavioral and somatic predictors of recurrent depressive symptoms, we tested the moderating effect of sex on the relationship between the specified predictors and recurrent depressive symptoms. Detailed results are displayed in Table 4. No sex-specific interaction term turned out to be significant. Only social support (OR = 0.93; 95% CI = 0.87 to 0.99) remained as single predictive factor for recurrence of depressive symptoms when taking all predictors including sex-specific interaction terms into account. Loneliness as risk factor failed to reach significance (OR = 1.61; 95% CI = 0.94 to 2.79).

**Table 4:** Multiple logistic regression model on recurrent depression (PHQ9 ≥ 10) for the overall sample (N = 797; N = 389 events).

	Odds Ratio	L (95%CI)	U (95%CI)	p	Standard error	z-value
***Sociodemographic***						
Sex	0.529	0.092	3.026	0.470	0.889	−0.717
Age [5 years]	0.948	0.818	1.097	0.480	0.075	−0.713
SES	0.967	0.913	1.024	0.250	0.029	−1.140
Living with partner	1.469	0.814	2.689	0.210	0.304	1.270
Age [5 years] x sex	0.976	0.811	1.176	0.800	0.095	−0.253
SES x sex	1.013	0.937	1.096	0.740	0.040	0.329
Living with partner x sex	0.878	0.417	1.834	0.730	0.377	−0.345
***Behavioral***						
Smoking	1.09	0.637	1.861	0.750	0.273	0.316
Active sports	1.048	0.631	1.741	0.860	0.258	0.180
Alcohol above tolerance	1.082	0.623	1.877	0.780	0.281	0.281
Smoking x sex	1.811	0.885	3.731	0.110	0.367	1.620
Active sports x sex	0.901	0.474	1.714	0.750	0.328	−0.317
Alcohol above tolerance x sex	1.455	0.681	3.123	0.330	0.388	0.968
***Somatic***						
CVD	1.093	0.553	2.165	0.800	0.347	0.257
Cancer	0.635	0.210	1.771	0.400	0.537	−0.847
Diabetes	0.945	0.432	2.052	0.890	0.396	−0.142
Obesity	1.213	0.713	2.069	0.480	0.271	0.712
CVD x sex	1.031	0.385	2.767	0.950	0.502	0.061
Cancer x sex	2.433	0.705	8.926	0.170	0.643	1.380
Diabetes x sex	0.597	0.204	1.738	0.340	0.545	−0.946
Obesity x sex	1.242	0.618	2.498	0.540	0.356	0.610
***Psychological***						
Type D personality	1.495	0.863	2.604	0.150	0.281	1.430
Life events (past 12 months) [per 5 events]	0.972	0.67	1.411	0.880	0.189	−0.149
Loneliness	1.614	0.935	2.792	0.085	0.278	1.720
**Social support**	**0.931**	**0.871**	**0.994**	**0.034**	**0.034**	**−2.120**
Generalized Anxiety	1.321	0.804	2.167	0.270	0.253	1.100
Social phobia	1.355	0.766	2.400	0.300	0.291	1.040
Panic	1.235	0.672	2.272	0.500	0.310	0.682
History of anxiety disorder	0.960	0.498	1.834	0.900	0.331	−0.123
Type D personality x sex	1.104	0.555	2.190	0.780	0.350	0.283
Life events (past 12 months) x sex	0.846	0.511	1.400	0.520	0.257	−0.650
Loneliness x sex	0.665	0.331	1.334	0.250	0.355	−1.150
Social support x sex	1.054	0.971	1.145	0.210	0.042	1.260
Generalized Anxiety x sex	0.958	0.507	1.812	0.890	0.325	−0.133
Social phobia x sex	0.779	0.379	1.599	0.500	0.367	−0.680
Panic x sex	1.127	0.521	2.445	0.760	0.394	0.304
History of anxiety disorder x sex	1.105	0.499	2.465	0.810	0.407	0.246
***Medication intake***						
Antidepressant	1.477	0.740	2.968	0.270	0.353	1.100
Antidepressant x sex	1.071	0.463	2.468	0.870	0.426	0.160

*Note*: PHQ-9 = Patient Health Questionnaire-9; SES = socioeconomic status; CVD = cardiovascular risk disease; L CI = lower confidence interval; U CI = upper confidence interval.

## Discussion

The purpose of this paper was to determine the prevalence of depressive symptoms in men and women at the five year follow-up in participants depressed at baseline based on a comprehensive data set from a large community sample. We took care to analyze data in a sex-sensitive way and included psychosocial and behavioral factors to capture potential gender effects. Aiming to close existing research gap regarding sex-specific effects of behavioral and somatic determinants of recurrent depressive symptoms, we comprehensively integrated social, psychological, behavioral and somatic predictors in our analysis models.

Almost half (48.4%) of depressed participants at baseline also reported elevated depressive symptoms five years later, with a higher, although not significant, trend among women. In univariate analyses with the entire sample, SES, smoking, antidepressant intake, and psychological variables (comorbid anxiety disorders, Type D personality, loneliness, and social support) were significant predictors. SES and social support were protective while the other characteristics constituted risk factors for recurrent depressive symptoms.

Univariate analyses stratified by sex indicated that SES, loneliness and social support were only predictive among men. Smoking was only a significant risk factor among women. Common risk factors were comorbid anxiety disorders, Type D personality and antidepressant intake. Subsequent separate multivariate analyses indicated different predictive patterns for recurrence of depressive symptoms. For men, only social support proved to be the single protective predictor. For women, unhealthy behavior, in particular smoking and a distressed personality were predictive for recurrent depressive symptoms.

Our findings corroborate previous research on predictors of repeated episodes of depression^3,16^, emphasizing the role of smoking and personality. In line with previous investigations of the gender gap in depression, individual differences such as negative affectivity, avoidance tendencies, and social inhibition especially predispose women to develop and sustain depressive symptoms. These aspects converged in a “developmental subtype” of depression, which was twice as often observed in women than in men, and have been postulated as one reason for the generally increased prevalence of depression in women^20^. Type D comprises the tendency to experience negative affect and to inhibit affective expression toward others. Negative affectivity and social inhibition^21^ have both been demonstrated as antecedents of depression. According to Kuehner^20^, negative affectivity scores of girls increase during adolescence and constitute a risk for the increased rate of depression in women compared to men. Goodwin and Gotlib^22^ and found that neuroticism was associated with increased vulnerability for depression in women but not in men.

Little is known about different functions and relevance of smoking for men and women’s mental health as previous research endeavors seldom carried out sex-specific analyses e.g.^23^. Emotion regulation has been identified as an important predictor of recurrence of depression^24^. Smoking, which has increased the risk for recurrent depression in our study, has been related to developmentally based emotion regulation difficulties after the experience of early adversities^23^. Consistent with our findings, two previous studies reported a stronger link between smoking and current and lifetime depression^25^ in women.

With regard to men, it is remarkable that only social support remained predictive of recurrent depressive symptoms considering the comprehensive model analyzed. Moreover, social support proved to be the only significant factor in the overall model, when sex-specific interaction terms were added to test moderating effects of sex. Hence, we cannot conclude that there are significant sex-differences regarding any identified predictive factors of recurrent depressive symptoms since no interaction term in the overall model reached significance. In conclusion, social support proved to be a protective factor against recurrence of depressive symptoms. Unlike previous studies, which did not differentiate concurrent from new onset depression^18^, especially men benefitted from perceived emotional and tangible social support^6^ compared to women regarding recurrent depressive symptoms. To a lesser extent, feelings of loneliness showed a trend for exacerbating the likelihood of recurrent depressive symptoms when taking all analyses results into account. These findings may inform the treatment of depressive symptoms.

Overall, our findings emphasize the crucial role of psychosocial components for the recurrence of depression^6,14^. Weak evidence for gender differences in the interplay of psychosocial and behavioral determinants was observed. Contrary to previous findings from a Dutch cohort study^8^, but similar to a multi-national study^19^, we did not find an association of chronic somatic diseases with recurrence of depressive symptoms. Findings underscore the importance of temporal dynamics in a recurrent or chronic course of depression. Factors precipitating new onset of depression, such as critical life events, may lose the significance to trigger further episodes of depression. Accordingly, predictors of incident depression^1^, such as comorbid anxiety disorders were no significant predictors of recurrent depressive symptoms any more in the multivariate models.

Insights in sex- and gender differences in pathways of depression are still lacking sound empirical evidence^9,20^. Although we used a large prospective cohort study as data base, cases of comorbidities were rather low in the final sex-stratified sample, i.e. for cancer in men. A selective drop-out within the time frame of five years between two measurement points due to weak physical constitution, especially among elderly, or severe psychological ill-health cannot be excluded. While we measured depression repeatedly, we had to rely on an - albeit valid - self-report scale. However, we did not assess the duration of depressive symptoms or episodes. Future studies may focus on tracking detailed mechanisms of trajectories in terms of remission and onset among depressed men and women. Due to the exploratory approach in this study and possible inflated type 1 error rates due to multiple testing, replications of with a confirmatory approach are highly recommended. The current study revealed predictors of recurrent depressive symptoms in women and men. Strengths refer to the large representative community sample and the comprehensive assessment of multiple sources integrating demographic, psychosocial, and behavioral determinants with mental and somatic comorbidities.

## Methods

### Procedure and study sample

The Gutenberg Health Study (GHS) is a population-based, prospective, observational single-center cohort study in the Rhine-Main-Region, Germany^26,27^. Its primary aim was to analyze and improve cardiovascular risk factors and their stratification. The study protocol and documents were approved by the ethics committee of the Medical Chamber of Rhineland-Palatinate (reference no. 837.020.07; original vote: 22.3.2007, latest update: 20.10.2015) and the local data safety commissioner. All study investigations were conducted in line with the Declaration of Helsinki and principles outlined in recommendations for Good Clinical Practice and Good Epidemiological Practice. Prior to enrolment, participants signed written, informed consent. The sample was drawn randomly from the local registry in the city of Mainz and the district of Mainz-Bingen, stratified 1:1 for sex and residence and in equal strata for decades of age. Inclusion criterion was age 35 to 74 years. Insufficient knowledge of German language, psychological or physical impairment with regard to participation led to exclusion. 5.2% were excluded based on these exclusion criteria. The response rate (defined as the recruitment efficacy proportion, i.e. the number of persons with participation in the baseline examination divided by the sum of number of persons with participation in the baseline examination plus those with refusal and those who were not contactable) was 55.5%.

At baseline, 15,010 participants were examined between 2007 and 2012. Of those, N = 12,061 filled out the PHQ-9 at two measurement (baseline and follow-up) points five years apart. Inclusion criterion for the current study was a PHQ-9 ≥ 10 at baseline indicating clinical relevant depressive symptoms. Thus, our analysis sample consisted of N = 877, age 52.3 (±9.9), 523 women (59.6%). In the following, we investigated the presence of depression (i.e. participants with relevant depressive symptoms who scored above 9 on the PHQ-9 at follow-up) in participants who were depressed at baseline.

### Materials and assessment

As the current work is a part of the ongoing cohort study in which study procedures, assessments and data management are highly standardized, the following descriptions of our main measures might correspond to previous publications, in particular^1^.

#### Measures

Sociodemographic variables were assessed via self-report: participants’ sex (1 = men, 2 = women), age in years, current employment (no/yes), whether they were currently living with partner (no/yes), and their socioeconomic status (SES). SES was defined according to Lampert, Kroll^28^ as ranging from 3 (lowest socioeconomic status) to 21 (highest socioeconomic status). The multidimensional index combines information about educational qualifications, household characteristics of occupation, and income with equal weights. The SES index can be categorized into three groups (low, medium and high) allowing for a comparison between the bottom and top 20% of the population with a broadly defined center comprising 60% of the population. In the present study, we used a continuous score.

#### Psychological measures

We used the PHQ-9 (Depression module of the Patient Health Questionnaire), a widely used screening instrument, in order to assess the presence of depressive symptoms at both measurement points (baseline and follow-up). The presence of clinically relevant symptom burden at follow-up was defined as a sum score ≥ 10. This cut-off has previously yielded good results with respect to internal consistency (in the present sample, Cronbach’s α was 0.80), sensitivity (81%), and specificity (82%) in detecting depressive disorders^29^.

We used the two-item short form of the Generalized Anxiety Disorder-7 GAD–7^30,31^; to assess symptoms of generalized anxiety. Its sum score (combining participants’ scores on both items) ranges from 0 to 6. Previous research has shown that a sum score of ≥3 detects the presence of generalized anxiety disorder with good sensitivity (86%) and specificity (83%)^30^.

We used the German version of the Mini-Social Phobia Inventory (Mini-Spin) to detect social anxiety. Its sum score ranges from 0 to 12. In previous research based on a representative sample of the German population, a cut-off score of 6 was able to separate individuals with generalized social anxiety disorder from controls with good sensitivity (89%) and specificity (90%)^32^.

Panic disorder was screened with the brief PHQ panic module. Corresponding to previous research, a clinically relevant level of symptom burden was present if participants answered at least two of the first four PHQ panic questions with ‘yes’^33^.

We assessed Type D (distressed) personality using the German version of the Type D Scale-14 (DS14) by Denollet^34^. The questionnaire comprises two reliable subscales with 7 items each. They assess negative affectivity (Cronbach’s α in the present sample was 0.88) and social inhibition (Cronbach’s α in the present sample was 0.86). Participants rate each item on a 5-point Likert scale (ranging from 0 = false to 4 = true). The presence of Type D was defined if participants scored 10 points or higher on both subscales.

We also used the Social Readjustment Rating Scale (SRRS) in order to assess the occurrence of potentially stressful life events. As a widely used checklist^35^, the SRRS provides researchers with a standardized measure of the impact of a wide range of different life events. In the context of the present study, we administered an adapted German version with 36 items. It showed good internal consistency (Cronbach’s α = 0.81). Participants rated life events depending on their occurrence over the past years (0 = no, 1 = yes). For analyses, we used the unweighted scores.

We assessed participants’ subjective experience of loneliness using a single item:’I suffer from frequently being alone /have few contacts’ which was rated on a Likert scale ranging from 0 to 4. The options were 0 = no, does not apply, 1 = yes it applies, but I do not suffer from it, 2 = yes, it applies, and I suffer slightly, 3 = yes, it applies, and I suffer moderately, 4 = yes, it applies, and I suffer strongly. In line with previous research, we recoded loneliness by combining 0 and 1 = no loneliness or distress; 2 = slight, 3 = moderate, and 4 = severe loneliness^36^.

In addition, we also asked for participants’ perceived social support using the Brief Social Support Scale BS6;^37^. The questionnaire consists of six items (3 per scale) which have previously assessed emotional and tangible support with good reliability (for the total scale, Cronbach’s α was 0.86). Participants were asked to rate the single items on a scale from 1 to 4 (1 = never, 2 = occasionally, 3 = mostly to 4 = always).

### Behavioral measures

Our measures of health behavior included smoking, which was assessed by self-report. Participants’ responses were dichotomized into non-smokers (=0; comprising never smokers and ex-smokers) and current smokers (=1; occasional smoker, i.e. cigarette/day, and smoker, i.e. cigarette/day).

Obesity was defined as a body mass index ≥30 kg/m². The binary variable was coded 0 = no obesity, 1 = obesity.

Alcohol consumption was also assessed by self-report and measured in grams per day. Increased alcohol consumption was defined in terms of daily consumption ≥24 g for men and ≥12 g for women which reflects the German threshold for alcohol consumption above tolerance.

We inquired physical activity with the Short Questionnaire to Assess Health-Enhancing Physical Activity SQUASH;^38^. The SQUASH captures physical activity in the context of commuting, leisure time, household, work and school activities. In line with previously established definitions, sleeping, lying, sitting, and standing were classified as physical inactivity^38^. Active sports was presented in quartiles with Q1 denominating the lowest and Q4 the highest quartile of physical activity.

### Interview assessments

As part of the computer-assisted personal interview (CAPI), participants were asked whether they had ever received a definite diagnosis of any depressive or anxiety disorder by a physician (in the following referred to as medical history of a lifetime diagnosis of any depressive, respectively anxiety disorder).

The presence of coronary heart disease was assessed by the question: ‘Were you diagnosed with a stenosis of your coronary vessels’ Self-reported myocardial infarction (MI), heart failure (HF), stroke, deep vein thrombosis (DVT), pulmonary embolism (PE), and peripheral arterial disease (PAD) were summarized as cardiovascular risk disease (CVD). Participants were also asked whether they had ever received a definite diagnosis of cancer by a physician.

Diabetes was defined in individuals with a definite diagnosis of diabetes by a physician or a blood glucose level of ≥126 mg/dl in the baseline examination after an overnight fast of at least 8 hours or a blood glucose level of >200 mg/dl after a fasting period of 8 hours.

Medications were registered on site at the GHS study center by scanning the bar codes from original packages of the drugs provided by participants. Current use (coded no = 0/yes = 1) of antidepressants was also assessed this way.

### Statistical analysis

Descriptive analyses were performed as absolute and relative proportions for categorical data, means and standard deviations for continuous variables and median with interquartile range if not fulfilling normal distribution. Inference tests between depression groups (no/yes) were calculated with t-tests or Chi^2^-tests.

In order to determine recurrence or remission of depressive symptoms at follow-up, we only included participants who were depressed (PHQ ≥ 10) at baseline. In order to identify predictors of recurrent depressive symptoms, we performed General Linear Models (GLM), in particular logistic regression analyses with depressive symptoms (PHQ-9 sum score ≥10) as the criterion at follow-up including sociodemographic, psychological, behavioral and somatic factors assessed at baseline as predictors. In order to identify specific effects in women and men, we first analyzed women and men separately. Subsequently, we tested interaction effects between sex and each of the specified predictors on recurrent depressive symptoms analyzing the entire sample. By conducting moderation analysis, we aim to determine significant sex-related differences in the specified predictors.

All p-values should be regarded as continuous parameters that reflect the level of statistical evidence, and they are therefore reported exactly. P < . 05, two-tailed, was considered significant. Statistical analysis was carried out using R version 3.3.1.^39^.

## Data Availability

The analysis presents clinical data of a large-scale population-based cohort with ongoing follow-up examinations. This project constitutes a major scientific effort with high methodological standards and detailed guidelines for analysis and publication to ensure scientific analyses on highest level. Therefore, data are not made available for the scientific community outside the established and controlled workflows and algorithms. To meet the general idea of verification and reproducibility of scientific findings, we offer access to data at the local database in accordance with the ethics vote upon request at any time. The GHS steering committee, which comprises a member of each involved department and the head of the Gutenberg Health Study (PSW), convenes once a month. The steering committee decides on internal and external access of researchers and use of the data and biomaterials based on a research proposal to be supplied by the researcher. Interested researchers make their requests to the head of the Gutenberg Health Study (Philipp S. Wild; philipp.wild@unimedizin-mainz.de). More detailed contact information is available at the homepages of the GHS (www.gutenberghealthstudy.org).
